# Cough Time Table

**Published:** 1887-02

**Authors:** J. E. Winans

**Affiliations:** Lyons Farms, N. J.


### COUGH TIME TABLE.

J. E. Winans, M. D., Lyons Farms, N. J.

**MORNING**—*Acon.*, Ail., ALUM., Amm. c., Amm. m., Ant. c., Apoc. and., *Arn.*, *Ars.*, *Arum d.*, Atrop., Aur., Bell., Bor., Brom., *Bry.*, *Calc. c.*, Carb. an., Carb. v., Caust., Cham., Chel., Chin., Chin. s., Cina, Coc. c., Crot. t., Cupr., Curar., Dig., *Dros.*, Dulc., *Euphr.*, Ferr., Grat., Gymn., Hep., *Iod.*, Ipec., *K. carb.*, K. bich., Kreos., Lachn., *Led.*, *Lyc.*, Mag. c., *Mag. s.*, MOSCH., Nat. ar., Nat. c., Nat. s., *Nat. m.*, Nitr., *Nux v.*, Ol. jec., Op., *Phos.*, Ph. ac., Phyt. (Ptel.), *Puls.*, Rhod., *Rhus t.*, Scilla., *Selen.*, Sep., Silic., Stann., Staph. (Stict.), Stram., Sulph., Sulph. ac., *Tabac.*, *Tarent.*, Tellur., Thuj., Verat. alb., Zinc, Zing.

—*Agg.:* Nux v., Stann.

—*Amel.:* Coc. c., Grat.

—**IN BED**—Amm. c., Bry., *Caust.*, *Coc. c.*, Ferr., *K. ca.*, Nitr., *Phos.*, Rhus t., Sep.

—**ON OR AFTER WAKING**—Ail., *Carb. v.*, Caust., *Coc. c.*, Cod., Ferr., *Ign.*, Mag. s., Nux v., Phos., Rhus t., *Rumex*, SILIC., Sulph., Tarent., Thuja.

—**AFTER RISING**—Alum., *Alumen.*, Amm. br., Ang., *Arn.*, Arg., Bary. c., Bov., *Carb. an.*, Carb. v., *China*, *Cina*, Dig., Euphorb., *Ferr.*, Lach., Nitr. ac., Nat. s., Paris., Sil., Staph., Sulph., Thuja.

—**AT BREAKFAST**—Senega, Alum.

**MORNING AND EVENING** (more especially)—Acon., Alumen, Bor., *Caust.*, Cina, Ign., *Lyc.*, Merc., Nat. m., Phos., *Rhus t.*, Sep., Silic., *Stram.*

**TILL EVENING**—Silic.

**DURING DAY**—Agar., Ail., Alum., Amm. c., *Anac.*, *Arg.*, *Arum d.* (BELL.), Brom., Calc. c., Chin. s., Cic. v., Coc. c., Coloc., Comoc., Con., Cotyl., EUPHR., Ferr. Gamb., Graph., Hepar. (Hydrst. c.), K. brom., Lach., Lauroc. (Lob. c.), *Lyc.*, Manc., Nat. ar., Nat. c., Nat. m., Nitr., Nitr. ac., Phos., (Puls. nut.), Sep., Silic., Sol. t. æg., *Spong.*, Stann., Staph., (Sticta), Sulph., *Sumb.*, Viola od.

**EVERY OTHER DAY**—*Anac.*, Lyc., Nux v., (Sep.?).

**DAY AND NIGHT**—Amm. m., Bell., Bism., Cupr., Dulc., Euphorb., Ign., Kalm., Lyc., Nat. m. (tickling), Nit. ac., Spong., Stann., Sulph. (spasmodic).

**FORENOON**—*Agar.*, Alum., *Aurum m.*, Bell., *Bry.*, *Camph.*, Canc. fl., Chin. s., Cocc. c., Grat., Hell., (K. carb.), Lact. v., Mag. c., Nat. ars., Nat. c., Rhus t., Sabad., Sars., Sep., Staph., Sulph. ac.

ONE A. M.—Coc. c., Sulph.

ONE TO TWO.—Zing.

ONE TO FOUR—Bufo.

TWO A. M.—(Amm. m.?) Chin., Chin. s., Cocc. (quartan.), *Dros.*, Glon., Nat.m., *Op.*, Phos., Rumex, Sulph.

—*Amel.* by drinking water—Glon.

TWO OR THREE A. M.—Ant. t., Merc. sol.

TWO TO THREE A. M.—Amm. c., K. bich.

TWO TO HALF-PAST THREE—Coc. c.

HALF-PAST TWO—(K. bich.)

TWO TO FIVE A. M.—Rumex.

MIDNIGHT TO THREE A. M.—*Acon.*

THREE A. M.—*Amm. c.*, Cainca, *K. carb.*, Mag. c., Mur. ac., Nitr., Op., Rhus t.

TOWARD THREE OR FOUR A. M.—*Bufo.*

THREE TO FOUR A. M.—Amm. c., *K. carb.*, Lyc.

(*Amel.*) AFTER THREE A. M.—Ant. t.

NIGHT, TILL FOUR A. M.—*Apis*, Nicc., Sil.

FOUR A. M.—*Anac.*, Asclep. t., China.

FIVE A. M.—Arum t., *Kali c.*, Rumex.

(*Agg.*), FIVE A. M. TO TWELVE M.—(K. carb.)

SIX A. M.—*Coc. c.*

SIX TO SEVEN A. M.—Arum t., Calc. ph.

SIX TO EIGHT OR NINE A. M.—*Cedron.*

SIX A. M. TO SIX P. M.—*Calc. ph.*

HALF-PAST SIX A. M.—Diosc.

SEVEN A. M.—Digitalin.

EIGHT A. M.—Diosc., Ham. v.

EIGHT TO NINE A. M.—Silic.

MORNING TILL NINE A. M.—Sep.

NINE A. M.—Sep., Tarent.

NINE TO TEN A. M.—Ars. hydg.

NINE A. M. TO TWELVE M.—Staph.

TEN A. M.—Aq. pet., Coc. c., Mag. c.

TEN TO ELEVEN A. M.—Coc. c., Sumbul.

ELEVEN A. M.—Raph., *Rhus tox.*

TOWARD NOON—*Silic.*

TWELVE M.—Agar., *Arg. n.*, *Arund.* (Bell.), Euphr., Naja, Staph., Sulph. (*Amel.*), *Mang.*

**IN AFTERNOON**, more especially—Agar., Amm. m., Anth. n., (Apis?) Arn., Bad., *Bell.*, Bov., Cepa, China, Coca, Fagop., Gamb., K. bich., Mag. c., Mezer., Mosch., Mur. ac., *Nat c.*, Nux v., Phell., *Phos.*, SANG., Staph., Sulph., Thuj., Zinc.

ONE P. M.—Nat. s.

ONE OR TWO P. M.—Aq. pet.

HALF-PAST ONE P. M.—Phallas.

TWO P. M.—Coca, Lauroc., Ol. an.

THREE P. M.—Ang., Calc. ph., *Coc. c.*, Hepar, Phell.

AFTER THREE P. M.—Canc. flv.

THREE TO FOUR P. M.—Calc. fl.

THREE TO TEN P. M.—Bell.

FOUR P. M.—Calc. fl., *Chel.*, Coca, K. bich., Saponin(?).

FOUR TO SIX P. M.—Lyc.

FOUR TO EIGHT P. M.—Lyc.

FOUR P. M. TO BED-TIME.—*Mang.*

FIVE P. M.—Cupr., Nat. m., Sol. t. æg.

FIVE TO NINE P. M.—Caps.

SIX P. M.—*Am. m.*, Chel., Physost. (?), *Rhus t.*, Sulph., Sumb. —(*Amel.*), from expectoration—Sulph.

SIX TO SEVEN P. M.—Ipec.

SIX TO TEN P. M.—Hyper.

QUARTER AFTER SIX P. M.—Ol. an.

SUNSET TO SUNRISE—(Aurum).

**IN P. M. AND EVENING** (more especially)—Amm. br., (Apis ?), Bell., *Lyc.*, *Nat c.*, *Phos.*, *Sang.*

**AT NIGHTFALL**—Eriodyct., Ign., Lyc., Puls.

**EVENING**—Act. r., Agn. c., Ail., Alum, *Ambra.*, *Amm. c.*, *Amm. m.*, Anac., Ant. c., Apis, Arg. n., Arn., *Ars.*, *Arum d.*, Arund., Aspar., Atrop., Bad., Bar. c., Bell., Bism., Bov., *Brom.*, Bry., *Calc.*, *Caps.*, Carb. an., *Carb. v.*, Caust., Cepa, Cham., Chel., China, Chin. s., Cina, Coc. c., Coff., *Con.*, Cop., Crot. t., *Dros.*, Dolich., Eug., *Eup. pf.*, Eup. pur., Euphr., Ferr., *Fluor. ac.*, Graph., Gymnoc., *Hepar*, Hcy. ac., Ign., Indig., *Iod.*, Ipec., K. bich., K. carb., K. jod., Kalm., Kreos., Lach., Lauroc., Led., Lith. c., LYC., Lycps., Mag. c., *Mag. m.*, Mar. v., Merc. v., Merc. jod. r., Mez., Morph., Mosch., Mur. ac., Naja, Nat. ar., Nat. c., Nat. m., *Nit ac.*, Nux m., *Nux v.*, Ol'nd., Ox. ac., Petr., Phell., PHOS., Ph. ac., Prun. sp., *Psor.*, *Puls.*, Rheum, *Rhus t.*, Rumex, *Sang.*, *Seneg.*, *Sep.*, Silic., *Sin. nig.*, Sol. t. æg., *Spong.*, *Stann.*, Staph., Sticta, *Still.*, Stront., *Sulph.*, Sulph. ac., Sumb.,

Tabac., Tarent. (Thasp. aur.), Thuja, *Verat.*, Verat v., *Verbas.*, *Zinc*, Zingib.

—**IN BED**—*Agn. c.*, ALUMN., AMM. C., Anac., Ars., Bell., *Calc. c.*, *Caps.*, Carb. v., Coca, Coff., Dolich., *Dros.*, Ferr., Graph., *Hep.*, Hyos., Ign., Indig., K. carb., Kreos., LYC., Mag. c., Mag. s., Merc., Nat. m., Nicc., Nux m., Nux v., Paris, Petr., Puls., Rhus t., Ruta, Sep., Staph., *Verbas.*, Thuja.

(*Amel.*) **IN EVENING**—Amm. m., Coc. c., Sulph., Zing.

WORSE IN EVENING TO TWELVE P. M.—Baryt. c., Bell., Caust., *Hepar*, Mez., Nux v., *Rhus t.*, Sep., Verat.

**BEFORE MIDNIGHT**—Apis, Cal. c., Carb. v., Ferr., Mez., *Nit. ac.*, Nux v., Osm., *Rhus t.*, Spong., *Stann.*

(*Amel.*) **BEFORE MIDNIGHT.**—Apis (q. v.).

SEVEN P. M.—Bry., Comoc., Grat., *Ipec.*, Iris foet., Spiran. (?), Spiræa (?).

SEVEN P. M. TO ONE A. M.—*Cainca.*

QUARTER AFTER SEVEN—Comoc.

HALF-PAST SEVEN P. M.—Act. r., Raph. (?).

SEVEN OR EIGHT P. M.—Sin. nig.

EIGHT P. M.—Diosc., Nat. m., Sep.

EIGHT TO NINE P. M.—Sep.

EIGHT TO ELEVEN P. M.—Nat. m.

TWENTY MINUTES AFTER EIGHT P. M.—Coca.

NINE P. M.—Apis, Diosc., *Silic.*

NINE P. M. to four A. M.—*Apis.*

**AT NIGHT** (including in bed) **MAINLY**—*Acon.*, Act. r., Alum., *Ambra*, AMM. BR., *Amm. c.*, *Am. m.*, Anac., Ant. t., Arg. n., Apis, Arn., *Ars.*, *Arum d.*, *Aur.*, *Bar. c.*, BELL., Bry., Cact. g., Calad., *Calc.*, *Caps.*, *Carb. an.*, *Carb. v.* (Card. m.), *Caust.*, *Cham.*, *China*, Cocc., Cocc. c., Cod., Coff., Colch., Comoc., Con.,

Coral., Cur., *Cycl.*, Dig., *Dros.*, Dulc., Eryng., Eugen., Eup. pf., Graph., *Grat.*, Hepar (Hydrst. c.), *Hyos.*, *Ign.*, *Ipec.*, Kali b., Kali c., Kalm., Kreos., Lachn., Lact., Led., LYC. (Lob. inf.), *Mag. c.*, *Mag. m.*, Mag. s., Meph., *Merc.*, *Mez.*, (Myrt. c.), Nat. m., Nicc., Nitr., Nit. ac., *Nux v.*, Ol. an., Ol. jec., Op., Paris, *Petr.*, Phell., Phos., Psor., *Puls.*, (Puls. nut.), Rhod., *Rhus t.*, *Rumex*, Ruta, Sabad., *Sang.*, Scilla, Seneg., Sep., *Silic.*, Spig., Stann., Staph., *Sticta*, Stront., *Sulph.*, Tarent., (Thasp. aur.), Therid., *Verat.*, *Verbas.*, Zinc, Zizia.

(*Agg.*) **AT NIGHT**—AMM. C., Anac., Ars., Arum d., Bell., Caps., *Cham.*, *Chel.*, Cod., Coloc., Cur., Gamb., *K. brom.*, *Nat. s.*, *Nit. ac.*, Op., Puls., *Stront.*, Tarent.

**EVERY OTHER NIGHT**—Merc. sol.

COUGH DURING SLEEP, OR AWAKING FROM DITTO—*Acon.*, Arn., *Bell.*, Calc. c., *Cham.*, Coff., Hippom., LACH., Merc., Nit. ac., Phos., Rhod., Sep., Sulph., Verbas.

TEN P. M.—*Bell.*, Diosc., Nat. m.

TEN P. M. TO ONE A. M.—Calad.

HALF-PAST TEN P. M.—Carbn. s., Sol. t. æg.

ELEVEN P. M.—*Ant. t.*, Arn., Baryt. c., *Bell.*, Carb. v., Caust., Ferr., Hep., Led., Lyc., Mag. c., Mag. m., Mosch., Mur. ac., Nit. ac., Puls., Rhus t., *Rumex*, Sabad., Sep., Stann., Staph., Sulph., Sul. ac., Verat., Zinc.

ELEVEN TO TWELVE P. M.—Hepar.

ELEVEN TO ONE A. M.—Cup. ac.

HALF-PAST ELEVEN P. M.—*Coc. c.*

TWELVE P. M.—(Amm. m.,) Ant. t., Bar. c., Bell., Calc. ac., *Cham.*, Cocc. (quartan.), Coff., Dig., Dros., Grat., K. carb., Mag. c., Mag. m., Manc., Naja, Nit. ac., Nux v. (lying on back), Phos., Ruta, Samb., *Sep.*, Sulph., Zing.

(*Amel.*)—Phos.—(sitting up), Sulph.—by lying on side—Nux v.

COUGH AFTER TWELVE P. M.—*Acon.*, Ant. t., *Ars.*, Arum d., Bell., Bry., *Calc. c.*, Cham., China, Dig., Dros., Hyos., K. carb., Mag. c., Merc., Mez., *Nux v.*, Samb.

