# Errata

**Published:** 1887-04

**Authors:** 


Errata.—Dr. Winans wishes us to make some corrections in his Cough Time Table under rubrics as follow: "Morning in bed" add *Nux v.* "Forenoon," *Amm. m.*, not Aurum m. "Half-past one P. M.," *Phellan*, not Phallas. This error may also be found in Allen's *Symptom Register*. "Twelve P. M., Amel.; sitting up" refers to Phos. "In P. M. and evening" add Sulph.

